# Feasibility Study of Signal Processing Techniques for Vibration-Based Structural Health Monitoring in Residential Buildings †

**DOI:** 10.3390/s25072269

**Published:** 2025-04-03

**Authors:** Fedaa Ali, Leila Donyaparastlivari, Seyed Ghorshi, Abolghassem Zabihollah, Mohammad Alghamaz, Alwathiqbellah Ibrahim

**Affiliations:** 1 Department of Electrical and Computer Engineering, The University of Texas at Tyler, 3900 University Blvd., Tyler, TX 75799, USA; fali3@patriots.uttyler.edu (F.A.); aghorshi@uttyler.edu (S.G.); 2 Department of Mechanical Engineering, The University of Texas at Tyler, 3900 University Blvd., Tyler, TX 75799, USA; ldonyaparastlivari@patriots.uttyler.edu (L.D.); malghamaz@patriots.uttyler.edu (M.A.); 3 Department of Mechanical, Environmental, and Civil Engineering, Tarleton State University, Stephenville, TX 76402, USA; azabihollah@tarleton.edu

**Keywords:** structural health monitoring, damage detection, signal processing, piezoelectric sensors, residential buildings, reliability

## Abstract

The growing vulnerability of residential buildings to seismic activity and dynamic loading underscores the need for robust, real-time Structural Health Monitoring (SHM) systems. This study investigates the feasibility of utilizing piezoelectric sensors integrated with advanced signal processing techniques, including Power Spectral Density (PSD) and Short-Time Fourier Transform (STFT), for vibration-based SHM in residential structures. A scaled three-story building prototype was fabricated and subjected to controlled base excitations at 25 Hz and 0.6 g acceleration to evaluate the response under three structural conditions: healthy, randomly damaged, and thickness-damaged. The experimental results revealed that structural degradation significantly altered sensor outputs, with random damage causing irregular signal dispersion and thickness damage introducing additional frequency harmonics. PSD analysis effectively identified shifts in energy distribution, signifying structural degradation, while STFT provided a detailed time-frequency representation, facilitating real-time damage detection. The findings confirm that piezoelectric sensors, when combined with PSD and STFT, can serve as a low-cost, scalable solution for early damage detection in residential buildings, offering a practical framework for enhancing structural resilience against earthquakes and other dynamic forces.

## 1. Introduction

Residential buildings in seismically active regions of the United States face persistent risks from earthquakes and other natural disasters, posing significant threats to structural integrity and occupant safety [1]. Seismic activity generates ground accelerations and vibrations that impose significant forces on buildings, frequently resulting in structural damage. Conventional mitigation strategies, such as seismic retrofitting, aim to enhance structural resilience against such forces [2]. However, effective damage reduction requires real-time assessment of building health to detect deterioration before catastrophic failure occurs. Additionally, accurately estimating potential losses due to earthquakes is essential for guiding mitigation efforts and minimizing long-term structural degradation.

Extensive research has been conducted to estimate earthquake-induced losses for various structural types [3,4,5]. However, beyond immediate damage, repeated exposure to seismic vibrations can lead to progressive microdamage, gradually weakening a structure over time. Although often invisible, this deterioration can result in sudden failure at forces well below the original design thresholds. Current evaluation methods, such as visual inspections, remain widely used due to their low cost and simplicity. However, these assessments are highly dependent on inspector expertise, prone to human error, and limited to discrete time intervals, leaving critical gaps in structural monitoring.

Structural Health Monitoring (SHM) systems offer a more advanced approach, enabling continuous, real-time assessment of structural performance, particularly during and after extreme events like earthquakes [6]. By leveraging early damage detection, SHM helps prevent catastrophic failures and provides timely alerts that can enhance safety measures. These systems typically consist of sensors, data acquisition units, communication networks, and centralized data processing platforms [7]. With advancements in computational power and sensing technologies, machine learning approaches have increasingly been integrated into SHM to improve real-time decision-making and anomaly detection [8,9,10].

Among the various types of sensors employed in SHM, piezoelectric sensors stand out as an effective and cost-efficient solution for real-time monitoring of structural integrity [11]. These sensors convert mechanical strain into electrical signals with high sensitivity, making them particularly well suited for capturing high-frequency vibrational data. Their application is especially valuable in regions prone to seismic activity, hurricanes, and other dynamic loading conditions, where structures experience rapid stress variations. By continuously capturing strain fluctuations, piezoelectric sensors facilitate the early detection of microcracks and material fatigue, allowing for timely interventions that can prevent catastrophic failure [12,13]. The effectiveness of piezoelectric sensors in detecting structural anomalies has been demonstrated in multiple studies, such as those by Ali et al. [14], and Park and Inman, who validated their use in damage detection through impedance-based monitoring techniques [15]. However, the reliability of these sensors depends significantly on their placement within the structure, necessitating optimization strategies to maximize their effectiveness [16].

To enhance damage detection capabilities, advanced signal processing techniques such as Short-Time Fourier Transform (STFT) and Power Spectral Density (PSD) are applied to the collected sensor data. Unlike traditional Fourier Transform (FT) or Fast Fourier Transform (FFT), which provide a static frequency representation, STFT enables continuous tracking of frequency content over time, making it particularly effective for monitoring non-stationary structural vibrations caused by progressive damage, environmental variations, or transient loads. This capability is particularly useful for detecting sudden structural changes, such as crack propagation or impact-induced damage. By mapping frequency variations over time, STFT can reveal the exact moments when these changes occur, an advantage that conventional FT and FFT techniques lack [17,18]. The time-frequency representation provided by STFT allows for a more comprehensive view of structural responses, enabling dynamic monitoring and precise anomaly detection [19,20].

By integrating these signal processing methods, SHM systems can detect both immediate damage and long-term structural deterioration by identifying subtle frequency shifts indicative of fatigue or microdamage [21]. PSD is particularly valuable for analyzing the frequency distribution of structural vibrations, providing insights into resonance and potential weaknesses [22,23]. STFT enhances time-frequency analysis, making it particularly useful for monitoring transient structural responses under dynamic loading conditions [24]. These techniques allow for a more precise and predictive understanding of structural behavior, improving both safety assessments and proactive maintenance strategies.

Fourier Transform (FFT), Power Spectral Density (PSD), and Short-Time Fourier Transform (STFT) each offer distinct analytical advantages in SHM. FFT provides a global frequency-domain representation, PSD quantifies the energy distribution across frequency bands, and STFT captures dynamic time-frequency interactions. When combined, these techniques significantly enhance the accuracy of damage detection in SHM systems. Studies have demonstrated the benefits of integrating these methods; for example, Wang et al. [25] showed that their combination improved damage detection in earthquake-exposed buildings by capturing both persistent and transient damage patterns. Similarly, Han et al. [26] found that applying FFT, PSD, and STFT together enhanced the monitoring accuracy of wind turbines, where complex environmental loads influence structural responses. Although this approach increases computational demand and requires expertise in interpreting multi-technique outputs, it substantially enhances damage detection reliability across varied structural conditions. Practical applications of these techniques include monitoring high-rise buildings for wind-induced fatigue using FFT and PSD [27] and detecting sudden frequency spikes associated with material failure in earthquake simulations through STFT [26]. Additionally, IoT-based SHM systems have been proposed for bridge health monitoring and real-time data collection, demonstrating their adaptability to various structural applications [28]. This study applies these well-established signal processing techniques to assess the structural integrity of residential buildings in hurricane- and earthquake-prone regions. While advanced methods such as wavelet transform and the Hilbert–Huang Transform (HHT) provide additional insights, this study emphasizes the practicality and reliability of FFT, PSD, and STFT for real-time, scalable, and accurate structural health monitoring.

This study presents a feasibility analysis of a low-cost yet efficient SHM system tailored for residential buildings in earthquake-prone areas. The proposed system integrates piezoelectric sensors to continuously monitor structural health by detecting voltage variations induced by deformation. Signal processing techniques, including PSD and STFT, are applied to analyze sensor data, offering deeper insights into structural behavior. A proof-of-concept experiment was conducted using an in-house experimental setup to simulate healthy, randomly damaged, and thickness-damaged structural conditions. The results demonstrate the potential of piezoelectric sensors in effectively monitoring and assessing structural damage, paving the way for scalable, real-time SHM solutions for residential buildings.

## 2. Residential Building Prototype Structure

**Figure 1:**
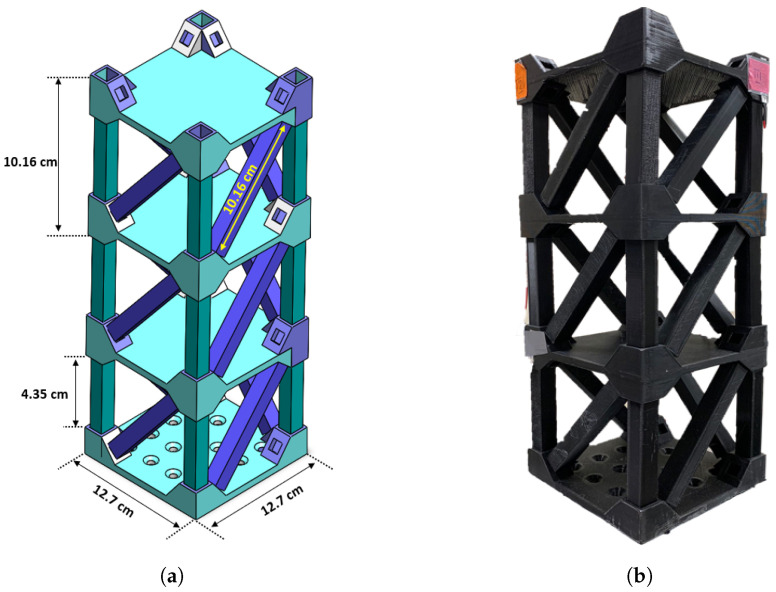
Multi -story building structure. (**a**) 3D schematic with dimensions, (**b**) 3D-printed PLA prototype.

To assess the performance and functionality of the proposed Structural Health Monitoring (SHM) system in residential buildings, a scaled 3D model of a three-story structure was designed and fabricated, as shown in Figure 1a. This prototype replicates key structural characteristics of modern multi-story residential buildings and serves as an experimental testbed for stability analysis. The model consists of three floors, including a ground floor and a base, with four primary vertical beams providing structural support. To evaluate its response to dynamic loading, the structure was subjected to base excitation to simulate seismic activity. To facilitate this testing process, grooves were integrated into the base to ensure secure attachment to an electrodynamic shaker.

The geometrical dimensions of the prototype, illustrated in Figure 1a, are 12.70 cm in length, 12.70 cm in width, and 40.00 cm in height. The structure was fabricated using polylactic acid (PLA), a widely used 3D-printing material recognized for its eco-friendly properties, ease of fabrication, and balanced mechanical performance. PLA provides sufficient strength and rigidity to maintain a stable experimental model while allowing for detailed structural replication.

To ensure a comprehensive understanding of the model’s structural properties, the geometric parameters of individual elements are specified as follows. Each column, beam, and diagonal brace features a square cross-section with a 1 cm side length. The nodal connections were 3D-printed with integrated slots to ensure consistent joint behavior. These geometric considerations were implemented to achieve a representative structural response under dynamic loading conditions. The finalized 3D-printed prototype is shown in Figure 1b.

In full-scale civil engineering structures, axial forces significantly influence strain distributions in columns, primarily due to self-weight and externally applied loads. However, in this scaled experimental model, bending effects are the dominant factor influencing structural response. While the present study prioritizes bending-induced strain analysis, it is acknowledged that in real-world applications, axial forces play a critical role, particularly in column stability. Column failure generally has more severe consequences than beam failure, making it a crucial aspect of structural health monitoring. Although this study focuses on bending effects, future work will integrate axial force contributions to provide a more comprehensive assessment of structural integrity.

## 3. Experimental Setup

**Figure 2:**
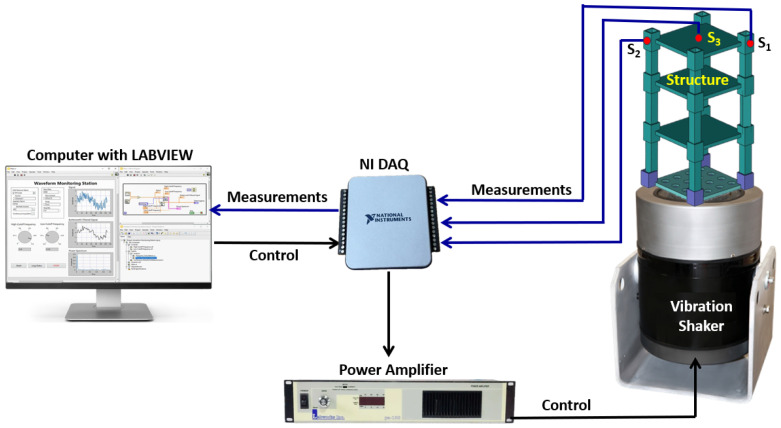
Schematic for the experimental setup. $S_{i}$ is the sensor location.

The experimental setup for the multi-story building structure, as illustrated in Figure 2, is designed to comprehensively investigate the structural response of the building to dynamic vibration applied to the foundations aimed to mimic the effect of earthquakes on the structural performance of the building. The primary components of this setup include three-story building models affixed to an electrodynamic shaker. This shaker is intricately linked to a power amplifier and seamlessly interfaced with LabVIEW software through an NI USB data acquisition board and a computer. This integrated setup enables precise control and monitoring of the vibrations experienced by the base of the three-story structure.

To capture detailed data on structural dynamics, an accelerometer manufactured by PCB Piezotronics with a sensitivity of 100 mv/g is placed at the base of the building to measure and monitor vibrations. The base is rigidly attached to the electrodynamic shaker, ensuring that the accelerometer accurately records the excitation applied by the shaker. This setup provides a precise understanding of the building’s response to dynamic loading conditions. Additionally, a network of piezoelectric sensors is strategically positioned across different sections of the three-story structure to capture its distributed response. These sensors, highlighted in red in Figure 2, are placed at various locations to ensure comprehensive coverage of structural behavior. Their distribution allows for representative sampling of vibrational responses, capturing localized variations in structural dynamics. By incorporating these piezoelectric sensors, the study enhances data granularity, facilitating a more detailed and reliable analysis of the building’s dynamic behavior under different loading conditions.

The placement of sensors in this study was chosen to maximize sensitivity to bending-induced strain, as the primary objective was to evaluate the feasibility of vibration-based structural health monitoring. Sensors were positioned at the top floor rather than on the columns because the flexural response of the structure is more pronounced at this location under base excitation. This allows for clearer signal differentiation between healthy and damaged conditions. However, it is acknowledged that axial strains in columns are critical in real structures, and future sensor placement strategies may incorporate column-mounted sensors to capture these effects more comprehensively.

## 4. Damage Detection Philosophy

**Figure 3:**
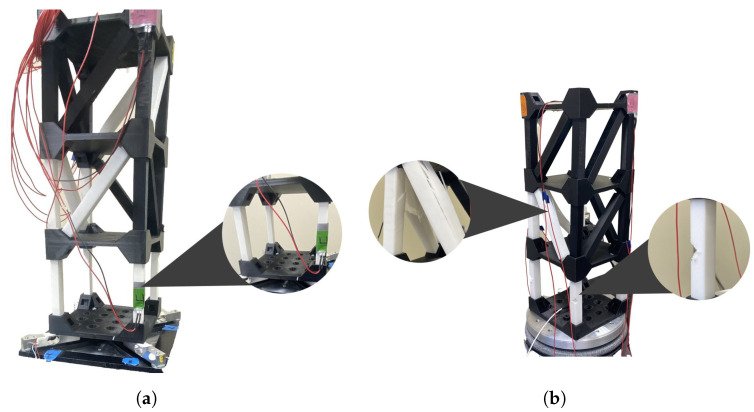
The structure conditions: (**a**) Thickness-damaged (**b**) Randomly damaged.

To evaluate the capability of the proposed Structural Health Monitoring (SHM) system in detecting structural damage, two distinct damage scenarios were introduced: element thickness reduction and random element failure. In the first scenario, selected structural elements were subjected to controlled reductions in thickness by introducing cuts, as illustrated in Figure 3a. In the second scenario, multiple elements were randomly damaged, including instances of catastrophic failure, as shown in Figure 3b.

Before assessing structural damage, the vibration response of the healthy structure was first determined and used as a baseline reference to quantify the severity of damage. The structure was securely mounted on the electrodynamic shaker, as depicted in Figure 2, and subjected to controlled excitations to analyze its dynamic behavior. Each damage scenario was tested three times, with variations in the damaged elements and severity levels, ensuring comprehensive evaluation of the monitoring system’s performance.

The experimental tests conducted to assess the effectiveness of the SHM system are summarized as follows:

1. Healthy Structure: The initial test was performed on the undamaged structure to establish a baseline response, as shown in Figure 1b.
2. Thickness-Damaged Structure: In this scenario, specific structural elements, particularly the columns, were intentionally weakened by reducing their thickness and infill density by 30% to simulate material degradation, as illustrated in Figure 3a.
3. Randomly Damaged Structure: The final test involved a structurally compromised model with randomly introduced cuts and breaks across multiple elements, representing an unpredictable damage pattern, as depicted in Figure 3b.

In this study, multiple identical structural models were used to evaluate the effects of different damage conditions. One model was preserved in its original state as a baseline for comparison (Healthy Structure), while two additional models were subjected to controlled modifications to simulate distinct damage scenarios. In the Random Damage Condition, structural elements, including beams and cross beams, were randomly cut or broken across 10 elements to replicate localized structural degradation. In the Thickness Damage Condition, selected elements were uniformly replaced with counterparts that were 30% thinner to simulate material loss and stiffness reduction. These modifications were applied to separate models to ensure that each test condition remained independent, preventing cumulative effects that could bias the results.

## 5. Results and Discussion

The Finite Element Model (FEM) and experimental investigation in this study serve complementary roles in evaluating the feasibility of vibration-based structural health monitoring. The FEM analysis provides critical insights into the structural response by predicting natural frequencies, mode shapes, and strain distributions under base excitation. These computational findings informed key aspects of the experimental setup, including sensor placement and expected vibrational behavior. However, while the FEM analysis identifies resonance conditions at higher natural frequencies, the experimental testing was deliberately conducted at lower excitation frequencies, where the structural response is significantly smaller in magnitude. This approach was chosen to rigorously assess the sensitivity of the piezoelectric sensors even in non-resonant conditions, demonstrating their capability to detect structural changes independent of resonance amplification. The ability to capture meaningful vibrational signals at sub-resonant frequencies underscores the robustness of the proposed monitoring system and highlights the effectiveness of the applied signal processing techniques, including the Power Spectral Density (PSD) and Short-Time Fourier Transform (STFT). This integrated framework ensures that the experimental findings validate not only the theoretical predictions but also the real-world applicability of the sensing methodology beyond idealized resonance scenarios.

### 5.1. FEA Modal Analysis

The dynamic stability of a structure is significantly influenced by its natural frequencies and mode shapes. When the frequency of external excitation approaches the natural frequencies of the building, the amplitude of vibrations can increase uncontrollably, potentially leading to catastrophic failure. Understanding these characteristics is crucial for developing effective monitoring and mitigation strategies.

**Figure 4:**
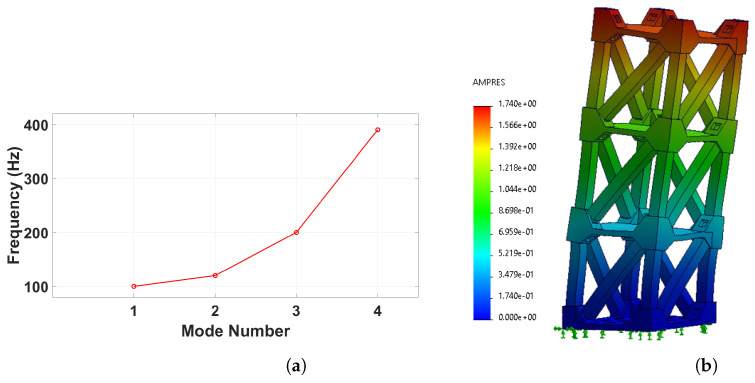
The frequency analysis simulation results: (**a**) Frequency mode plot, (**b**) First mode.

To evaluate the dynamic response of the structure, a finite element analysis (FEA) was conducted using the mechanical simulation software SolidWorks-2023 to determine the natural frequencies and corresponding mode shapes [14]. The first mode shape, in particular, provides valuable insights for selecting optimal sensor placement in Structural Health Monitoring (SHM) applications. The results of the analysis reveal the first four natural frequencies of the structure, as presented in Figure 4a.

The first natural frequency was identified as 111.8 Hz, with its corresponding mode shape illustrated in Figure 4b. As observed, the highest deflection occurs at the top floor of the structure. However, while deflection is highest in this region, the induced strain may not necessarily follow the same distribution. It is well established that strain concentrations are often more pronounced near fixed support points. However, since the foundations are designed to withstand high loads, strain measurements in these regions are less critical for the purpose of this study. Instead, to effectively demonstrate the feasibility of the monitoring system, strain measurements were taken near the top floor, where structural deformations are more visually evident.

Accordingly, three piezoelectric sensors were strategically positioned on the structure: two at the edges of the upper floor and one at the center of the top floor. These sensor locations, chosen to capture the structural response in regions of significant deformation, are illustrated in Figure 1b.

### 5.2. Signal Processing Analysis

This study aims to investigate the feasibility of utilizing piezoelectric sensors for structural health monitoring by conducting a proof-of-concept experiment. To assess their effectiveness in detecting structural damage, experimental testing is performed under controlled conditions with a base excitation frequency of 25 Hz and an amplitude of 0.6 g. The selected excitation parameters provide a consistent framework to evaluate sensor response across different structural conditions, demonstrating their potential for real-time monitoring and damage detection.

The sensor outputs exhibit distinct differences when comparing the healthy structure, the randomly damaged structure, and the thickness-damaged structure under identical base excitation conditions.

**Figure 5:**
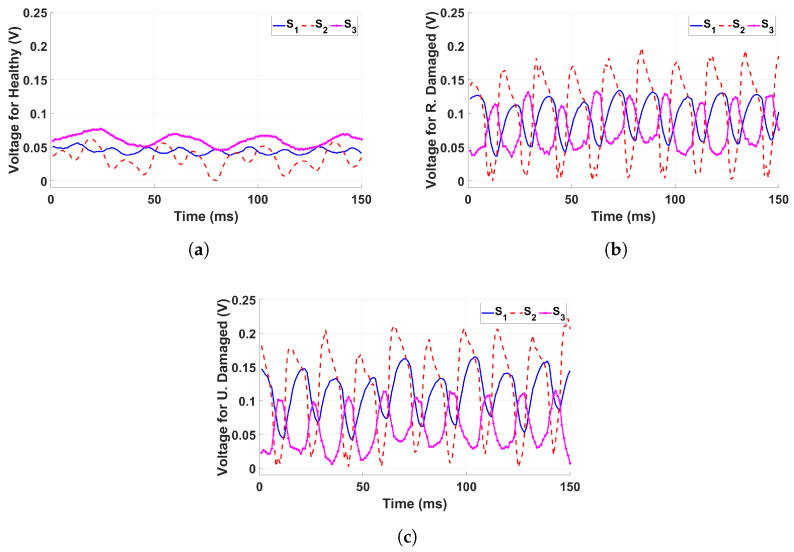
The voltage signals from each sensor, recorded under a 25 Hz base excitation at 60.48 mV (0.6 g), are presented for three test conditions: (**a**) Healthy condition, (**b**) Randomly damaged condition, and (**c**) Thickness-damaged condition.

In the healthy structure (Figure 5a), all three sensors ($S_{1},S_{2},S_{3}$) produce relatively smooth and periodic signals with consistent waveforms. The voltage amplitudes remain low and stable, indicating a uniform structural response to the applied 25 Hz excitation frequency and 60.48 mV amplitude. The minimal variation between sensor outputs suggests that the structure maintains its integrity, distributing vibrational energy predictably.

In contrast, the randomly damaged structure (Figure 5b) exhibits significant deviations in sensor outputs. The waveforms become irregular, particularly for Sensor Two ($S_{2}$), which displays pronounced oscillations and sharper peaks. The random nature of the structural damage results in an unpredictable signal distribution, causing each sensor to respond to varying stress concentrations throughout the structure. The increased signal amplitudes and irregularities suggest a highly localized and uneven stress response due to the randomly induced cuts and breaks.

For the thickness-damaged structure (Figure 5c), the voltage signals indicate moderate yet noticeable fluctuations, particularly in Sensors Two and Three ($S_{2},S_{3}$), which exhibit increased oscillation amplitudes compared to the healthy condition but remain lower than those observed in the randomly damaged scenario. This behavior reflects the non-uniform stiffness reduction caused by thinning elements, leading to localized stress concentrations without the complete unpredictability seen in the randomly damaged case.

Overall, these results confirm that sensor readings effectively capture structural degradation patterns, distinguishing between uniform vibrational responses in an undamaged state and the irregularities introduced by controlled structural modifications.

**Figure 6:**
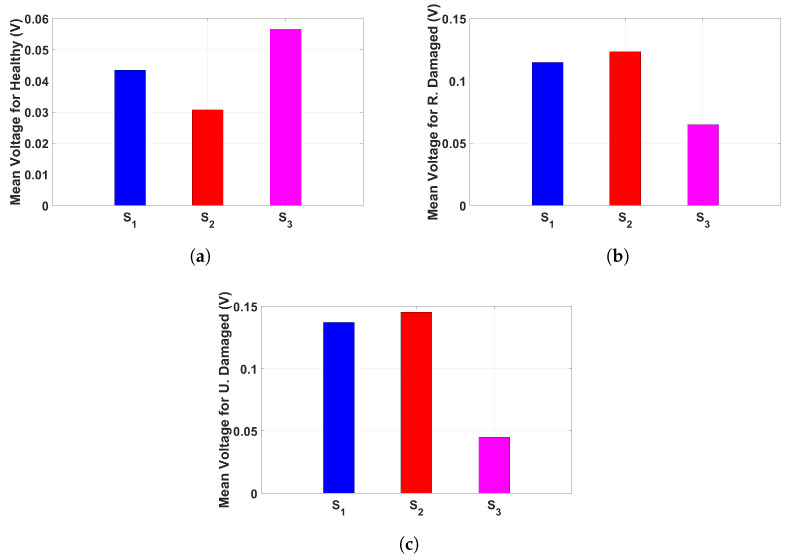
The mean voltage output from each sensor, recorded under a 25 Hz base excitation at 60.48 mV (0.6 g), are presented in bar plots for three test conditions: (**a**) Healthy condition, (**b**) Randomly damaged condition, and (**c**) Thickness-damaged condition.

Figure 6 presents the mean voltage output from three sensors under different structural conditions when subjected to a 25 Hz base excitation at 60.48 mV (0.6 g). The results, visualized through bar plots, highlight the variation in sensor response across the tested conditions, providing insights into the relationship between structural damage and voltage output. Under the healthy condition, shown in Figure 6a, the voltage output varies across the three sensors, with Sensor 3 exhibiting the highest response, followed by Sensor 1 and then Sensor 2. This suggests that even in an undamaged state, strain distribution is not entirely uniform, likely due to sensor placement and inherent structural properties. When the structure experiences random damage, as depicted in Figure 6b, a significant increase in voltage is observed in Sensors 1 and 2, while Sensor 3 records a lower response compared to the other two. This indicates that localized structural damage results in higher strain concentrations in specific regions while reducing the overall response in other areas. In the case of thickness damage, illustrated in Figure 6c, Sensors 1 and 2 exhibit the highest voltage readings among all conditions, while Sensor 3 experiences a significant drop in output compared to the other cases. This suggests that the uniform reduction in material stiffness redistributes strain in a manner that amplifies deformation in certain locations while reducing it in others. The observed trends confirm that piezoelectric sensors effectively capture variations in structural response due to different types of damage. These results reinforce the capability of the monitoring system in detecting and distinguishing between damage conditions, highlighting its potential for real-time structural health monitoring applications. The voltage signals from each sensor were separately analyzed to evaluate their behavior across three structural conditions: healthy, randomly damaged, and thickness-damaged structures. This examination provides crucial insights into signal distribution and structural integrity.

**Figure 7:**
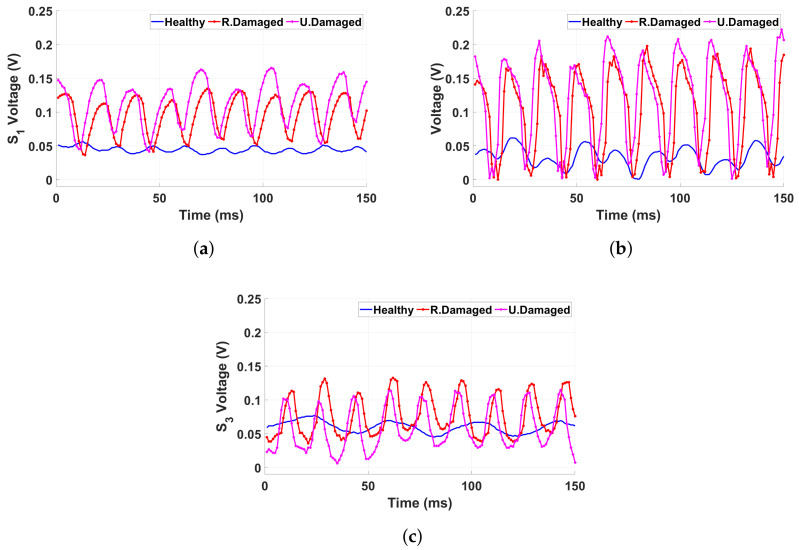
The voltage signals from each sensor, recorded under a 25 Hz base excitation at 60.48 mV (0.6 g), are presented for three test conditions of healthy, randomly damaged, and thickness-damaged from (**a**) Sensor 1, (**b**) Sensor 2, and (**c**) Sensor 3.

Sensor 1, as shown in Figure 7a, exhibits a low and consistent voltage response in the healthy structure, reflecting minimal strain-induced variations. Under the thickness-damaged condition, voltage peaks increase significantly, suggesting a more structured and amplified response due to the uniform reduction in material stiffness. In contrast, when the structure is randomly damaged, the voltage output reaches its highest and most irregular peaks, indicating chaotic signal distribution caused by unpredictable damage patterns. Similarly, as shown in Figure 7b, Sensor 2 exhibits a comparable trend. In the healthy condition, it produces a stable, low-voltage response. With thickness damage, the voltage peaks become more pronounced, indicating localized strain concentrations. Under random damage, the signal becomes increasingly erratic, exhibiting sharp voltage fluctuations due to stress redistribution in structurally compromised regions. Sensor 3, presented in Figure 7c, also demonstrates a consistent voltage response in the healthy condition. Voltage peaks increase under thickness damage, but its response to random damage appears slightly less chaotic compared to Sensors One and Two. This suggests that different sensor placements influence sensitivity to specific types of structural degradation. The overall sensor responses highlight the distinct effects of damage types on signal behavior. The randomly damaged structure produces the most unpredictable and erratic voltage outputs, whereas the thickness-damaged structure shows more systematic but elevated signals. The healthy structure maintains smooth, low-voltage signals across all sensors, confirming its stability. These findings emphasize the capability of piezoelectric sensors to detect and differentiate damage conditions based on voltage variations.

**Figure 8:**
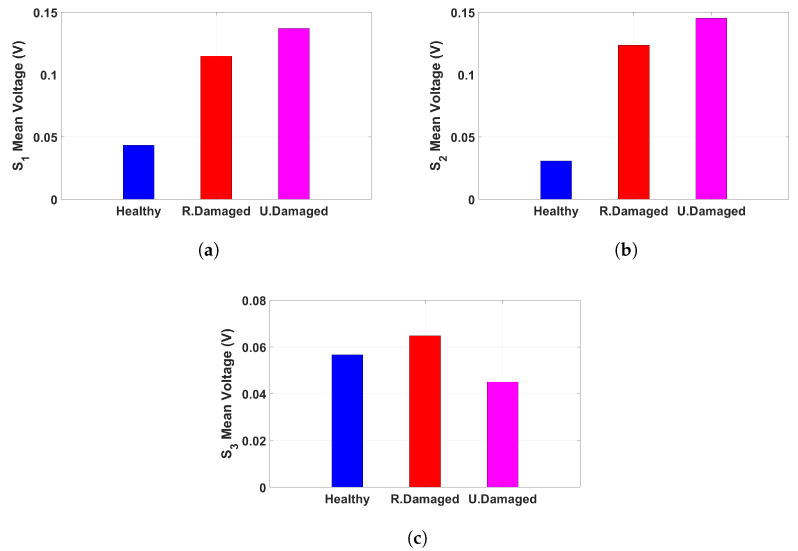
The mean voltage output recorded under a 25 Hz base excitation at 60.48 mV (0.6 g) presented in bar plots for three test conditions of healthy, randomly damaged, and thickness-damaged from (**a**) Sensor 1, (**b**) Sensor 2, and (**c**) Sensor 3.

Figure 8 presents the mean voltage output recorded from each sensor under different structural conditions when subjected to a 25 Hz base excitation at 60.48 mV (0.6 g). The results, visualized in bar plots, illustrate the correlation between structural integrity and sensor response. Across all sensors, a consistent trend emerges, showing increased voltage outputs under damaged conditions compared to the healthy state. Figure 8a,b demonstrates that sensors positioned in different locations exhibit similar trends, with the highest voltage observed in the uniformly damaged condition, followed by the randomly damaged case. This suggests that structural modifications significantly impact strain distribution, which is effectively captured by the piezoelectric sensors. However, Figure 8c reveals a slight deviation, where the randomly damaged condition exhibits a higher voltage than the uniformly damaged case. This indicates that sensor placement plays a critical role in damage detection, as certain locations may be more sensitive to specific types of structural degradation. The findings confirm that piezoelectric sensors can effectively monitor structural changes by detecting variations in voltage output. The distinct response patterns reinforce the potential of these sensors for real-time structural health monitoring, enabling early detection of localized damage and contributing to enhanced safety assessments.

The Power Spectral Density (PSD) results provide a detailed assessment of the structural response by quantifying the distribution of signal power across different frequencies under varying conditions. This analysis is critical for identifying resonant frequencies and understanding how energy is dissipated or redistributed due to structural modifications. Additionally, the Short-Time Fourier Transform (STFT) results offer a time-frequency representation of the signals, enabling the tracking of transient changes in the structural response over time. These combined analyses allow for a comprehensive evaluation of the sensors’ performance and their ability to capture variations in structural integrity.

**Figure 9:**
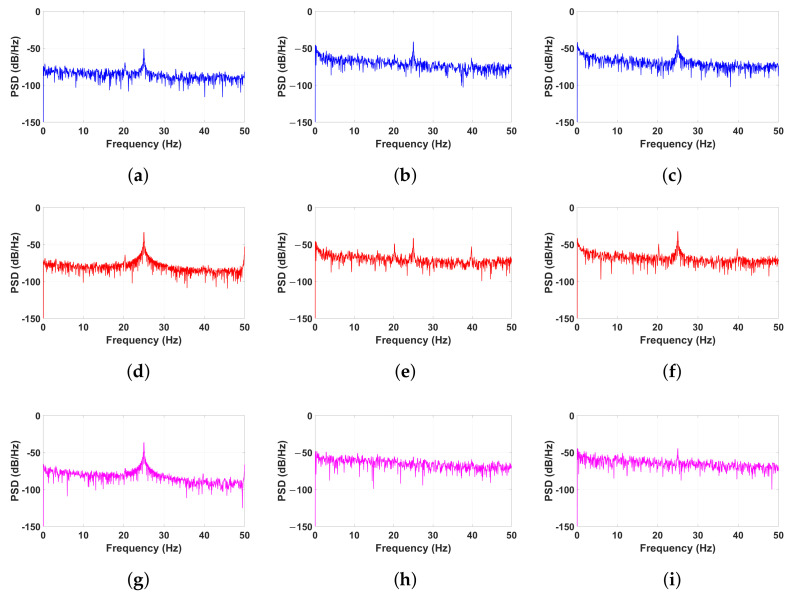
Illustration of the results of PSD for all sensors with a fixed amplitude of 60.48 mV at a frequency of 25 Hz in healthy (**a**,**d**,**g**), random (**b**,**e**,**h**), and thickness (**c**,**f**,**i**) damage conditions.

The PSD results obtained from the three sensors reveal distinct variations across the three tested conditions: healthy, randomly damaged, and thickness-damaged, as shown in Figure 9.

In the healthy condition (Figure 9a,d,g), all sensors exhibit a well-defined peak at the input excitation frequency of 25 Hz. This strong peak indicates that the majority of the vibrational energy is concentrated at the excitation frequency, reflecting the structure’s ability to respond efficiently with minimal energy dissipation. The absence of significant power distribution at other frequencies suggests structural stability and uniform vibrational behavior. The clear, isolated peak at 25 Hz confirms that the structure remains undisturbed by external noise or irregular frequency components, reinforcing its integrity.

In contrast, the randomly damaged condition (Figure 9b,e,h) exhibits significant alterations in power distribution. Instead of being confined to 25 Hz, the vibrational energy spreads across multiple frequencies, creating a broader spectrum. This dispersion indicates that structural damage disrupts the uniform energy concentration, leading to unpredictable vibrational behavior. The increased spectral noise and the reduced prominence of the 25 Hz peak suggest that random fractures and element failures introduce additional frequency components, reducing the structural coherence and stability.

In the thickness-damaged condition (Figure 9c,f,i), the primary excitation frequency at 25 Hz persists, though additional higher-frequency components become apparent. This suggests that while the structure retains some of its original vibrational properties, the reduced material stiffness introduces new harmonic responses and secondary vibrational modes. The presence of increased spectral content at higher frequencies implies a deviation from the pure resonance observed in the healthy state. Notably, Sensor 2 (Figure 9f) displays a more complex frequency response, indicating localized weaknesses where vibrational energy is distributed inefficiently. These additional frequency peaks highlight areas of concern where the structure’s ability to maintain a stable resonance is compromised.

The overall findings confirm that the PSD analysis effectively differentiates between the three structural conditions by identifying variations in frequency distribution and power concentration. The results demonstrate that structural damage leads to significant changes in vibrational energy dispersion, with random damage introducing the most erratic response, while thickness reduction results in modified but still detectable frequency shifts. These findings reinforce the capability of piezoelectric sensors and PSD analysis in detecting structural degradation, making them valuable tools for real-time structural health monitoring.

Comparing the three sets of results, the healthy structure exhibits the most stable and consistent sensor response, indicating uniform vibrational behavior. In contrast, the thickness-damaged structure introduces moderate irregularities, suggesting localized changes in stiffness and energy distribution. The randomly damaged structure, however, displays the highest level of signal variability, reflecting the unpredictable and non-uniform nature of the damage. These findings demonstrate how different damage types influence piezoelectric sensor outputs, with random damage causing more erratic and less predictable signal fluctuations compared to the more systematic deviations observed in the thickness-damaged case.

**Figure 10:**
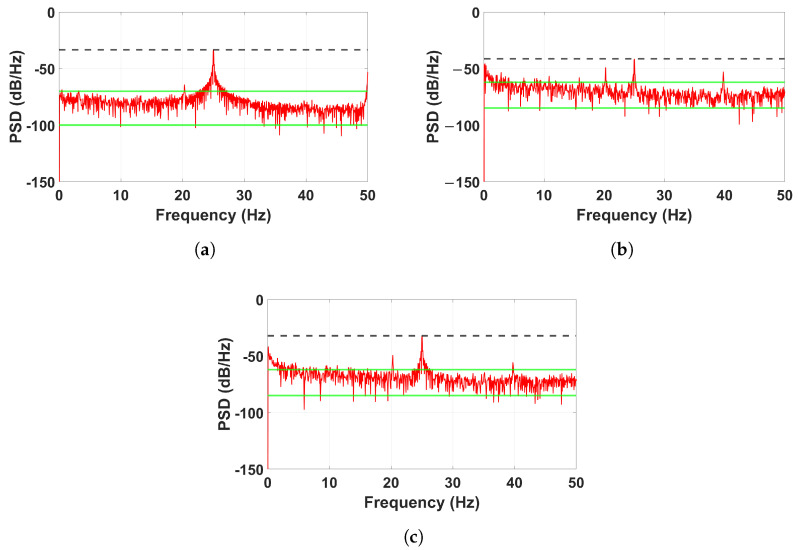
Power spectral density (PSD) comparison of Sensor 2 Signal in (**a**) Healthy condition, (**b**) Random damage condition, and (**c**) Thickness damage condition. The black dashed line indicates the upper power threshold, while the green lines represent lower baseline thresholds. These references help visualize deviations in vibrational energy across different damage conditions.

While Figure 9 provides a comprehensive overview of the power spectral density (PSD) results across all sensors, Figure 10 focuses specifically on a single sensor (Sensor 2) to offer a more detailed perspective on how structural damage alters the frequency response. By isolating the response of one sensor, we can better observe localized variations in frequency power distribution under different conditions. The use of dashed black markers and solid green threshold indicators enhances the understanding of how energy shifts across frequency bands due to structural degradation, emphasizing the sensitivity of piezoelectric sensors in detecting vibrational changes. These results further validate the effectiveness of PSD in identifying structural health conditions and distinguishing between different types of damage.

In the healthy condition (Figure 10a), the PSD plot exhibits a well-defined peak at 25 Hz, corresponding to the base excitation frequency. The concentration of vibrational energy at this frequency, with minimal dispersion, indicates a stable structural response where the structure efficiently transmits energy without significant losses or irregularities. The dashed black threshold line marks the upper power level, while the solid green threshold line establishes a lower bound, highlighting that the power remains concentrated around 25 Hz with minimal noise outside this range.

In contrast, the randomly damaged condition (Figure 10b) reveals a noticeable redistribution of energy. The intensity of the 25 Hz peak is significantly reduced, and energy spreads across a broader frequency range. This behavior suggests that random cuts and breaks disrupt the uniformity of the structure, leading to irregular vibrational modes. The dashed black threshold line, which previously captured the well-defined peak in the healthy condition, now shows a more erratic power distribution, indicating increased structural instability. Additionally, the solid green threshold line reveals that energy levels at lower frequencies have increased, further confirming the presence of new vibrational components induced by structural irregularities.

For the thickness-damaged condition (Figure 10c), the 25 Hz peak remains visible, but additional frequency components and noise emerge at higher frequencies. These secondary peaks suggest that the modifications to the structure introduce harmonics and secondary vibrational modes, altering its natural frequency response. The dashed black threshold line indicates an expansion of power distribution beyond the 25 Hz peak, while the solid green threshold line captures increased baseline fluctuations, reflecting the structure’s reduced ability to sustain a pure vibrational mode. This increase in frequency content highlights the complex resonance behavior induced by the reduced thickness of structural elements.

**Figure 11:**
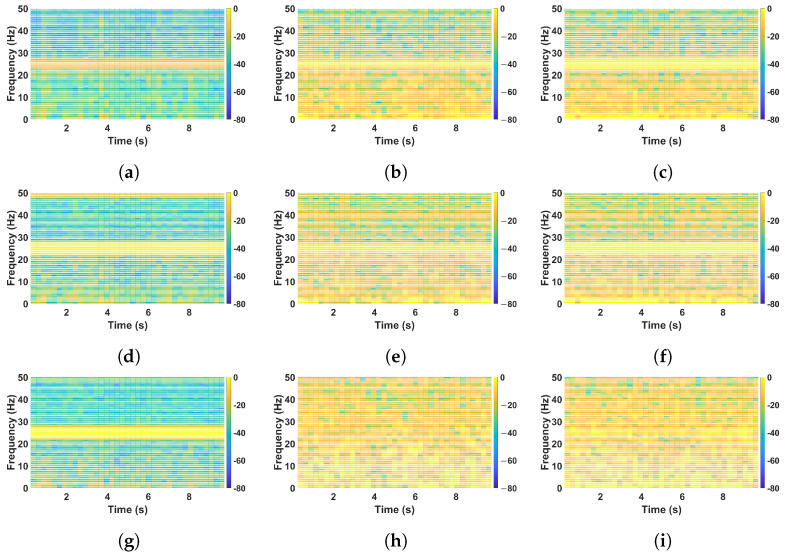
Illustration of the results of STFT for all sensors with a fixed amplitude of 60.48 mV at a frequency of 25 Hz in healthy (**a**,**d**,**g**), random (**b**,**e**,**h**), and thickness (**c**,**f**,**i**) damage conditions.

The Short-Time Fourier Transform (STFT) results in Figure 11 provide a detailed time-frequency representation of the vibration signals from all three sensors under healthy, randomly damaged, and thickness-damaged structural conditions. This visualization enables the identification of dominant frequency components, their persistence over time, and the presence of broadband energy that may indicate noise or irregular vibrational behavior.

For the healthy condition (Figure 11a,d,g), the spectrograms display a clearly defined and narrow bright band centered at approximately 25 Hz, which corresponds to the input excitation frequency. This bright and continuous region across time reflects a stable and coherent vibrational response. The surrounding regions remain relatively dark, indicating minimal energy spread across other frequencies and minimal background noise. This pattern is typical of a structurally sound system with strong resonance and minimal disruption.

In contrast, for the randomly damaged condition (Figure 11b,e,h), the STFT plots show a moderate dispersion of vibrational energy. While the 25 Hz component is still visible, it appears less sharp and more fragmented. Additional intermittent bright spots are observed at various frequencies and times, reflecting the introduction of irregular secondary vibrational modes due to random cuts and structural discontinuities. However, these changes are localized and occur in a somewhat structured manner, suggesting altered dynamics rather than a generalized increase in background noise.

The thickness-damaged condition (Figure 11c,f,i) shows a distinctly different behavior. Although the 25 Hz resonance is still present, the overall spectrogram appears smeared with a widespread presence of low-to-moderate intensity energy across a broad frequency range and throughout the duration of the signal. This results in a less defined contrast between the dominant frequency and the rest of the spectrum. Unlike the randomly damaged case, where localized frequency shifts are prominent, the thickness-damaged plots exhibit more continuous and elevated background brightness, particularly in Figure 11f,i. This pattern indicates a general elevation in vibrational energy across frequencies, which is interpreted as increased noise due to reduced material stiffness and a loss of structural damping. This persistent broadband energy is a key distinction from the randomly damaged condition and suggests that noise, defined here as power not localized to a specific frequency, is more dominant in the thickness-damaged state.

In summary, while both damage types introduce complexity to the vibrational response, the thickness-damaged condition is characterized by a higher level of distributed spectral energy, indicating elevated noise levels. This distinction underscores the importance of STFT in revealing nuanced differences in structural behavior and validating the piezoelectric sensor system’s capability to discriminate between varying damage scenarios.

The STFT results demonstrate that in the healthy condition, vibrational energy is well confined to the input frequency, ensuring a stable response. In the randomly damaged condition, energy dispersion and additional frequency components indicate chaotic structural behavior. In the thickness-damaged condition, the presence of harmonics and noise reflects a more complex and less efficient resonance pattern. These findings confirm the effectiveness of STFT in identifying changes in structural integrity and capturing damage-induced variations in frequency content.

To further clarify the scope of this study, it is important to emphasize that this work primarily serves as a proof-of-concept to demonstrate the feasibility of utilizing piezoelectric sensors and signal processing techniques, specifically Power Spectral Density (PSD) and Short-Time Fourier Transform (STFT), for real-time structural health monitoring in residential buildings. The primary objective is to establish that distinct vibrational characteristics associated with different structural conditions can be effectively captured and analyzed using these techniques. At this stage, the visual interpretation of frequency and time-frequency patterns remains a widely used approach in early feasibility studies, as it allows for a clear differentiation between structural conditions without requiring extensive statistical modeling. While additional quantitative statistical measures, such as confidence intervals and hypothesis testing, could further strengthen the analysis, their inclusion falls beyond the scope of this initial investigation. Instead, this study lays the groundwork for future research, which can build upon these findings by incorporating advanced statistical modeling and uncertainty quantification for more comprehensive validation. To reflect this, the discussion and conclusion sections explicitly state that the current study focuses on establishing feasibility rather than conducting a full-scale statistical validation and that future investigations will explore quantitative statistical measures to further validate and refine the methodology. This structured approach ensures that the study remains aligned with its intended objective while providing a strong foundation for continued research in this area.

## 6. Future Work

While Finite Element Models (FEMs) provide valuable insights into structural behavior, they alone cannot fully determine the optimal sensor placement due to the unpredictable nature of external forces such as earthquakes, wind loads, and other natural disasters. The complexity of these dynamic events requires an approach that integrates both computational modeling and optimization techniques to ensure effective sensor deployment. Various sensor placement optimization algorithms, such as genetic algorithms, particle swarm optimization, and convex optimization methods, have been developed to maximize monitoring efficiency while minimizing sensor redundancy. These techniques allow for the identification of optimal sensor locations based on predefined objectives, such as maximizing sensitivity to structural changes or ensuring comprehensive spatial coverage. Future studies could explore the integration of such optimization frameworks to enhance the reliability of vibration-based structural health monitoring systems.

## 7. Conclusions

This study demonstrated the feasibility of utilizing piezoelectric sensors in combination with signal processing techniques for vibration-based structural health monitoring in residential buildings. Experimental testing under controlled conditions showed that structural changes caused by random damage and thickness reduction produced measurable differences in the sensors’ time-domain and frequency-domain responses. Power Spectral Density (PSD) analysis revealed changes in energy distribution, with random damage leading to dispersed vibrational energy and less coherent resonance, while thickness damage introduced additional harmonics and broader frequency content. The Short-Time Fourier Transform (STFT) further illustrated how these structural changes evolved over time, capturing transient shifts and identifying variations in dominant frequency components. While the frequency content and power varied depending on the type of damage the structure was exposed to, it is important to emphasize that these techniques primarily detect the presence and evolution of structural changes rather than pinpointing their exact type, location, or severity. Nonetheless, the observed differences across damage scenarios confirm the sensitivity of the proposed method to distinguish general failure modes based on spectral patterns. Importantly, the effectiveness of this system does not rely on high-excitation sources. The results suggest that similar patterns can be captured using low-amplitude or ambient vibration sources, making this approach both practical and scalable. Overall, the findings validate that piezoelectric sensors, when paired with PSD and STFT, provide a reliable, real-time, and low-cost solution for monitoring structural behavior. This study offers a strong foundation for future implementation of SHM systems tailored for residential infrastructure, with potential to enhance resilience against seismic events and other dynamic loads.

## Data Availability

Data available on request from the authors.
